# Oocyte maturation and pregnancy outcomes in relation to gonadotropin duration in antagonist cycles

**DOI:** 10.1080/07853890.2026.2705793

**Published:** 2026-07-25

**Authors:** Hong Zeng, Shuyi Li, Jing Zhao, Nenghui Liu, Yanping Li

**Affiliations:** a Department of Reproductive Medicine Center, Xiangya Hospital, Central South University, Changsha, Hunan, China; b Clinical Research Center for Women’s, Reproductive Health in Hunan Province, Changsha, Hunan, China

**Keywords:** Gonadotropin stimulation duration, mature oocyte rate, GnRH antagonist protocol, *in vitro* fertilization and embryo transfer

## Abstract

**Objective:**

Gonadotropin (Gn) stimulation duration is a modifiable factor in antagonist protocols, yet its association with oocyte maturation and pregnancy outcomes remains unclear. This study aimed to determine whether Gn duration is independently associated with oocyte maturation and pregnancy outcomes after controlling for key confounders and to identify an optimal Gn window in GnRH antagonist cycles with fresh embryo transfer.

**Methods:**

This retrospective study included 9372 GnRH antagonist cycles at Xiangya Hospital. Multivariable regression models adjusting for confounders were used to assess the associations of Gn duration with oocyte maturation and pregnancy outcomes. Subgroup analyses were stratified by ovarian response. Generalized additive models were employed to identify nonlinear trends, followed by segmented and LOESS regression to determine the optimal duration.

**Results:**

After comprehensive adjustment, Gn duration maintained a significant nonlinear association with oocyte maturation (adjusted OR 1.07, 95% CI 1.05–1.09, *p* < .001), plateauing at approximately 9 days. Each additional stimulation day before 9 days was associated with increased maturation odds (adjusted OR 1.11, 95% CI 1.06–1.17; *p* < .001), while extended duration beyond 9 days showed no significant benefit (adjusted OR 0.94, 95% CI 0.88–1.01; *p* = .08). This association was absent in very low responders (≤5 oocytes) but consistent across other response subgroups. The 8–10 day duration window was associated with the highest likelihood of pregnancy and live birth.

**Conclusion:**

In GnRH antagonist cycles, Gn duration is associated with oocyte maturation and pregnancy outcomes following fresh embryo transfer, even after adjusting for key confounders. The 8–10 day Gn window represents a range associated with the highest likelihood of clinical pregnancy and live birth. These findings support prioritizing dynamic, response-adapted stimulation protocols aimed at achieving trigger criteria within this window, rather than adhering to fixed duration targets.

## Background

Assisted reproductive technology (ART) has undergone remarkable advancements, with controlled ovarian stimulation (COS) established as a cornerstone of successful reproductive outcomes following fresh embryo transfer. The introduction of gonadotropin-releasing hormone antagonist (GnRH-ant) protocols in the 1990s represented a significant breakthrough, offering distinct advantages such as shorter treatment duration, a reduced risk of ovarian hyperstimulation syndrome (OHSS) and improved patient tolerance [1]. Despite these benefits, the treatment success rates following fresh embryo transfer continue to be influenced by multiple COS parameters, among which the duration of gonadotropin (Gn) stimulation remains a critical yet underexplored factor.

Gn stimulation duration is pivotal in coordinating follicular development, oocyte maturation and endometrial receptivity. Inadequate stimulation periods may lead to asynchronous follicular development, potentially resulting in nuclear maturation arrest or cytoplasmic immaturity – both of which can compromise fertilization potential and embryo quality [2]. However, some clinical studies have reported no significant correlation between shorter stimulation duration and pregnancy outcomes [3,4], a discrepancy that may reflect the substantial interpatient variability in ovarian response.

Conversely, prolonged Gn stimulation may adversely affect invitro fertilization (IVF) outcomes through multiple mechanisms. Preclinical studies indicate that sustained high levels of follicle-stimulating hormone (FSH) can accelerate nuclear maturation while increasing the incidence of chromosomal abnormalities in oocytes [5]. Supporting human data demonstrate a dose-dependent rise in granulosa cell aneuploidy following extended Gn exposure [6]. Complete oocyte maturation necessitates synchronization of nuclear and cytoplasmic processes; while nuclear maturation can be assessed morphologically, cytoplasmic maturation involves complex functional changes that are not readily visible [7]. Furthermore, prolonged stimulation can desynchronize the endometrial window of implantation, particularly when progesterone elevation occurs prior to trigger – a well-documented factor impairing embryo-endometrial synchrony [8–10]. Additional evidence from murine models suggests that Gn administration downregulates vascular endothelial growth factor (VEGF) expression in endometrial tissues during the peri-implantation period, potentially compromising embryonic development [11]. These findings collectively underscore the delicate balance required in determining optimal Gn stimulation duration to support oocyte competence while maintaining endometrial synchrony.

Current evidence regarding optimal Gn duration remains inconclusive. Most available data derive from GnRH agonist long protocols, with studies reporting conflicting outcomes. While some reports found comparable pregnancy rates across different stimulation durations [4,12], others observed inferior outcomes with prolonged stimulation [13]. Findings from agonist protocols may not be directly applicable to antagonist cycles due to their distinct pharmacological mechanisms. GnRH antagonist regimens generally require 1–2 fewer stimulation days than agonist protocols [14] and some evidence suggests they may yield marginally lower pregnancy rates [15]. Patient-specific characteristics introduce additional complexity, as individuals with obesity [16] and polycystic ovary syndrome (PCOS) [17] often require longer stimulation yet exhibit reduced success rates. Similarly, poor ovarian responders demonstrate different follicular recruitment patterns compared to normal responders [18]. Interestingly, in antagonist cycles, poor responders who achieved pregnancy had shorter stimulation durations than their normal-responder counterparts. For normal responders, stimulation lasting fewer than 6 days may be insufficient for complete oocyte maturation, leading to reduced oocyte maturation rates (OMRs) and compromised pregnancy outcomes [19].

Despite its clinical importance, the relationship between Gn duration and treatment outcomes in GnRH antagonist cycles remains poorly defined, particularly regarding its complex interaction with underlying ovarian response. This study therefore aimed to determine whether Gn duration is independently associated with oocyte maturation and pregnancy outcomes after adjusting for key confounders and to identify an optimal Gn duration window in antagonist cycles with fresh embryo transfer.

## Materials and methods

**Figure 1.**
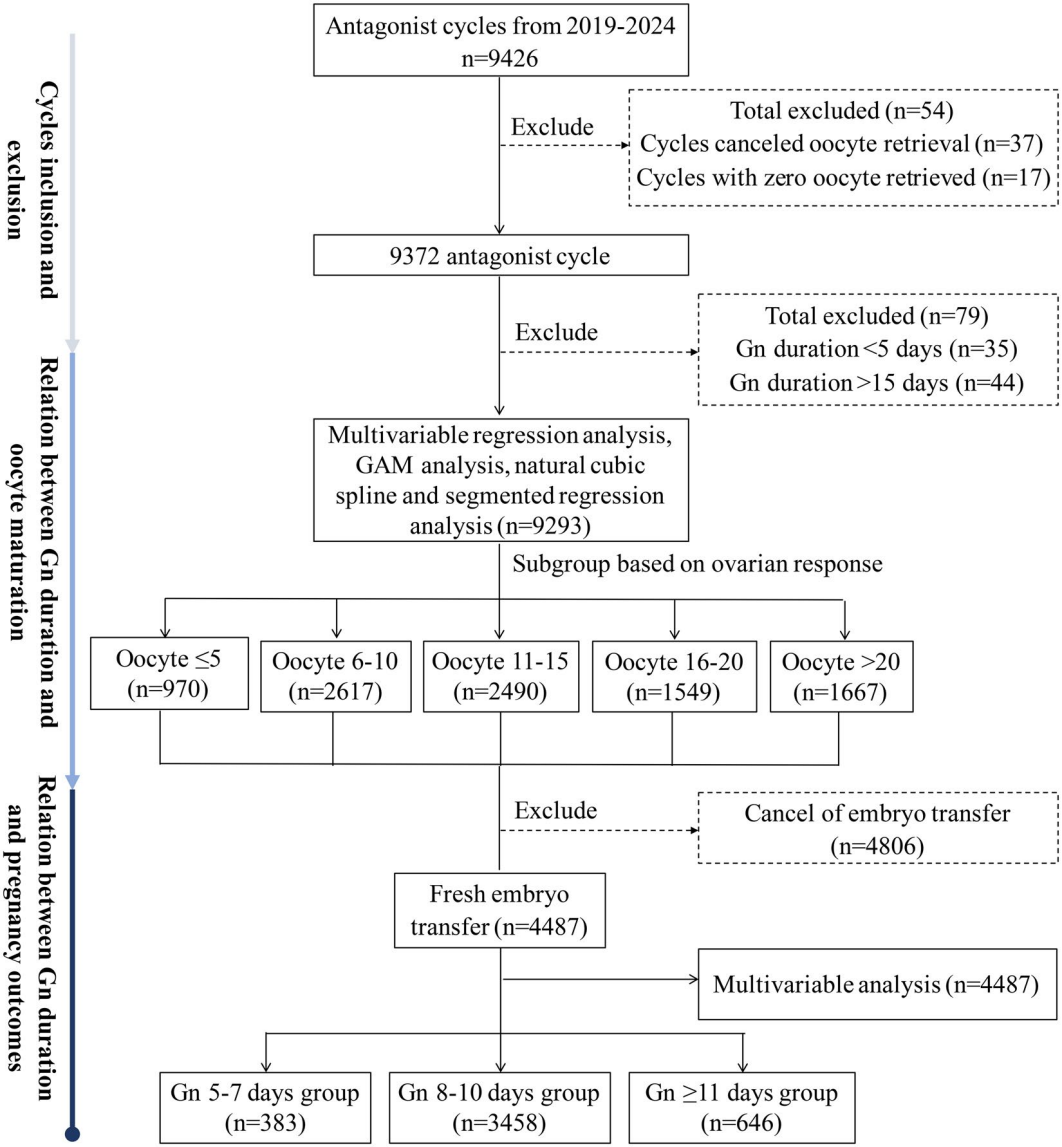
Flow chart of retrospective study.

This retrospective cohort study included stimulation cycles using a GnRH antagonist protocol at the Reproductive Medicine Center of Xiangya Hospital between October 2019 and July 2024. Patients’ data were extracted from electronic medical records. A total of 9426 GnRH antagonist cycles were initially considered during the study period. After excluding cycles cancelled before oocyte retrieval (*n* = 37) and cycles with no oocytes retrieved (*n* = 17), 9372 cycles proceeded to oocyte retrieval and were included for the primary outcome analysis of oocyte maturation. From these, cycles with extreme Gn durations (<5 days, *n* = 35; >15 days, *n* = 44) were further excluded due to small sample sizes and highly variable outcome trends, resulting in 9293 cycles for the primary analysis of the relationship between Gn duration and oocyte maturation. For the secondary analysis of pregnancy outcomes, the sample was restricted to cycles that underwent fresh embryo transfer (*n* = 4487). A detailed flow chart of patient selection and exclusion is presented in Figure 1. The study was approved by the Ethics Committee of Xiangya Hospital (Approval number: 2025005). The primary objective was to examine the association between Gn stimulation duration and oocyte maturation, as well as pregnancy outcomes.

### Controlled ovarian stimulation (COS) protocol

Ovarian stimulation was initiated on menstrual cycle day 2 using recombinant follicle-stimulating hormone (rFSH). The starting Gn dose was individualized based on patient characteristics, including age, body mass index (BMI), anti-Müllerian hormone (AMH) level and antral follicle count (AFC). Ovarian response was monitored *via* transvaginal ultrasound and serum hormone assessments. GnRH antagonist (cetrorelix, 0.25 mg; Merck-Serono, Geneva, Switzerland) was introduced on stimulation day 5 or when the leading follicle reached 14 mm in diameter. In our centre, all cycles followed a standardized trigger protocol: (1) trigger was considered when ≥2 follicles reached ≥18 mm or ≥3 follicles reached ≥17 mm with human chorionic gonadotropin (hCG; 6000–10,000 IU; Livzon Corp., Shanghai, China); (2) for patients at high risk of OHSS (with many follicles and high E_2_ level), the hCG trigger dose was reduced (4000–6000 IU) or a GnRH agonist (0.1–0.2 mg) was used for triggering; (3) for patients with prior empty follicle syndrome or poor embryo quality in previous IVF/intracytoplasmic sperm injection (ICSI) cycles, a higher hCG dose with extended trigger-to-oocyte retrieval interval or dual trigger (hCG + GnRHa) was considered. No planned early or delayed triggering was implemented during the study period to minimize variability in clinical decision-making.

### Oocyte retrieval and evaluation

Oocyte retrieval was conducted 35–36 h after trigger administration using 17-gauge single-lumen needles (Cook Medical, USA) without follicular flushing. All follicles with diameter >10 mm were retrieved. Retrieved oocytes were immediately assessed for nuclear maturity under ×200 magnification using an inverted microscope (Nikon, Tokyo, Japan). Oocytes were classified into three maturity stages according to standard morphological criteria: germinal vesicle (GV, immature with visible germinal vesicle), metaphase I (MI, intermediate, with germinal vesicle breakdown but no polar body) and metaphase II (MII, mature, with polar body but no germinal vesicle).

### Luteal phase support and embryo transfer

Luteal phase support was initiated on the day of oocyte retrieval and consisted of 200 mg oral progesterone daily plus 600 mg vaginal progesterone (Utrogestan, Besins Healthcare, France). Embryo transfer was performed on either day 3 (cleavage stage) or day 5 (blastocyst stage). Embryo quality was assessed continuously from day 2 to day 5. Good-quality cleavage-stage embryos were defined as grade I or II embryos with 7–9 blastomeres on day 3, according to the Istanbul consensus criteria. Good-quality blastocysts were defined as those scoring ≥3AA, 3AB, 3BA, or 3BB based on Gardner’s grading system.

### Outcomes definitions

The primary outcome is the OMR, calculated as the number of MII oocytes divided by the total number of oocytes retrieved. The secondary outcomes are pregnancy outcomes including biochemical pregnancy rate per embryo transfer (ET) cycle, clinical pregnancy rate per ET cycle, miscarriage rate per clinical pregnancy and live birth rate per ET cycle. Biochemical pregnancy was defined as a serum hCG level >10 IU/L 12 days after embryo transfer. Clinical pregnancy was confirmed by ultrasound visualization of one or more gestational sacs at 28–35 days post-transfer. Miscarriage was defined as the loss of a clinical pregnancy before 24 weeks of gestation. Live birth was defined as the delivery of any live infant after 24 weeks of gestation.

### Statistical analysis

Categorical variables are presented as frequencies and percentages and continuous variables as means with standard deviations (SD) or medians with interquartile ranges (IQR), as appropriate. Group comparisons were performed using the χ^2^ test for categorical variables. For continuous variables, the Student’s t-test or Mann-Whitney U test was used for comparisons between two groups and one-way ANOVA or Kruskal–Wallis test was used for comparisons among three or more groups, depending on the data distribution. Post-hoc analyses were conducted for multi-group comparisons. The quasi-binomial generalized linear regression was used to analyse factors associated with oocyte maturation. ORs and 95% CIs were estimated using quasibinomial generalized linear models with a logit link, with the response specified as the number of MII oocytes and the number of non-MII oocytes (total retrieved oocytes minus MII oocytes). To investigate factors associated with oocyte maturation, we constructed a series of multivariable models with incremental adjustment: Model 1 adjusted for Gn duration, AMH level and BMI. Model 2 additionally adjusted for E2 on hCG day and Progesterone level on hCG day. Model 3 further adjusted for female age, FSH level on hCG day and LH level on hCG day. Finally, variables with adjusted p values consistently <0.1 in the three models were included in model 4. Model 4 included Gn duration, AMH, E2 level on hCG day and Progesterone level on hCG day. Therefore, Model 4 was defined as the final parsimonious model with adjusted p-values of all included variables < 0.01. To investigate the nonlinear association between gonadotropin (Gn) duration and oocyte maturation, we adopted a sequential analytical strategy. First, a generalized additive model (GAM) was fitted using the mgcv package in R, incorporating a penalized smoothing spline for Gn duration along with AMH, oestradiol on hCG day (E2‑hCG) and progesterone on hCG day (P4‑hCG) as covariables. A quasibinomial family with logit link was used to account for overdispersion in the proportion of mature oocytes. Statistical significance of the smooth term (effective degrees of freedom >1 and *p* < .05) confirmed a nonlinear relationship. Next, to obtain a parametric threshold estimate, we applied a natural cubic spline (ns function, splines package) with 2 degrees of freedom (selected by AIC), which places a single internal knot at the median of Gn duration. The knot location served as a candidate transition point. Finally, segmented regression (segmented package) was performed to validate this breakpoint and estimate the change in the odds of oocyte maturation before and after the optimal cutoff, with adjustment for the same covariables. This combined approach ensured robust detection and quantification of the nonlinear threshold effect. Locally estimated scatterplot smoothing (LOESS) curves were applied to the raw data to visualize the unadjusted trends in both the absolute number of mature oocytes and the oocyte maturation rate across Gn duration. Subgroup analyses were performed based on the number of retrieved oocytes. Multivariable regression was further applied to evaluate the association between Gn duration groups and pregnancy outcomes. All statistical tests were two-sided with *p* < .05 considered statistically significant and .05 ≤ *p* < .10 was considered indicative of a trend towards significance. Analyses were performed using R version 4.5.1 with the ‘mgcv’ (v1.9-1), ‘segmented’ (v2.1-0), splines (v4.5.1) and ‘compareGroups’ (v4.8.0) packages.

## Results

### Study population characteristics

**Figure 2.**
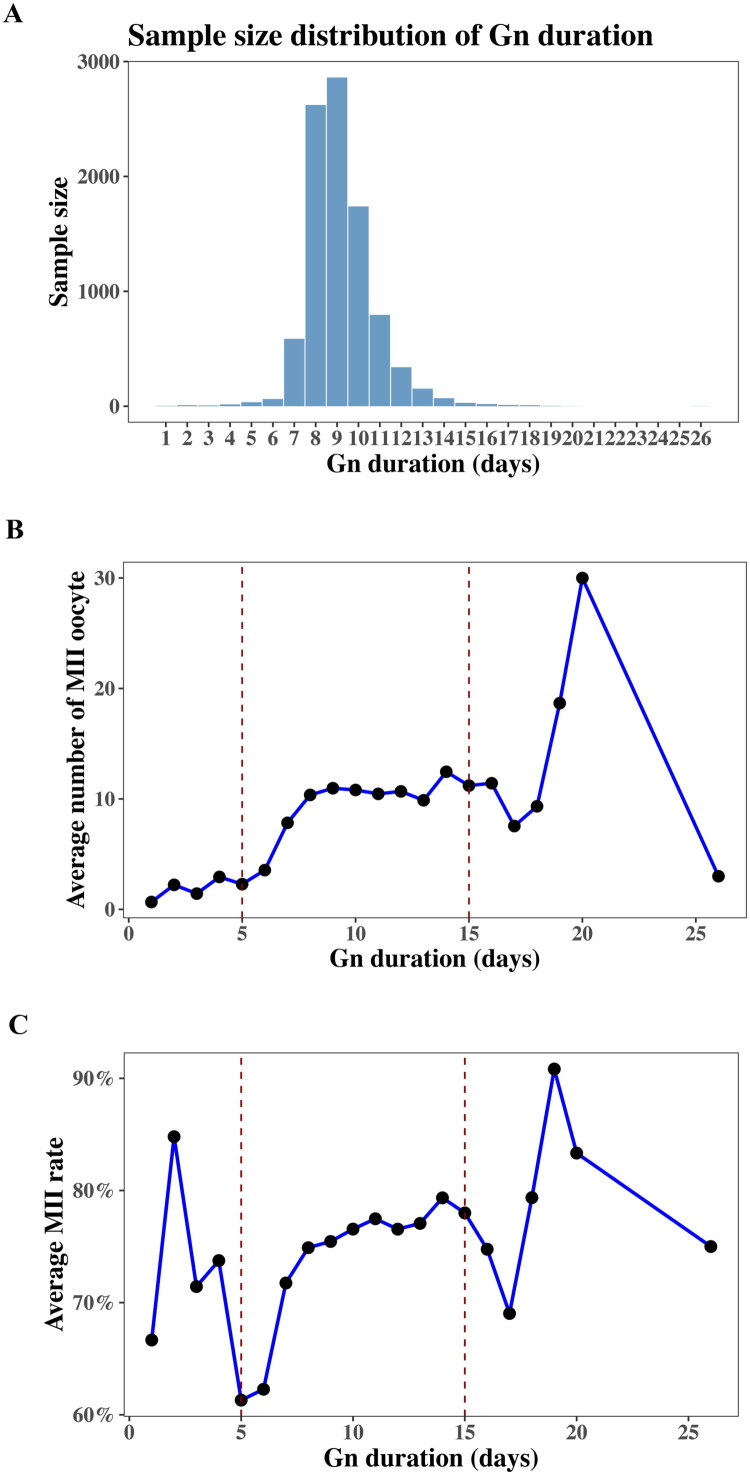
Distribution of gonadotropin (Gn) stimulation duration and its relationship with oocyte maturation outcomes. (A) Frequency distribution of cycles across different Gn duration days. (B) Line plot showing the average MII oocyte number across different Gn duration days. (C) Line plot showing the average MII oocyte rate across different Gn duration days. Gn: gonadotropin.

This analysis included 9372 GnRH antagonist cycles that proceeded to oocyte retrieval and yielded at least one oocyte during the study period. The distribution of gonadotropin stimulation duration is presented in Figure 2(A), with the majority of cycles (99.16%) falling within the 5–15 day range. Notably, cycles with Gn duration <5 days (*n* = 35) typically involved patients with diminished ovarian reserve who presented with pre-existing dominant follicles on cycle day 2, allowing rapid fulfilment of trigger criteria after only 3–4 days of stimulation. Conversely, cycles with Gn duration >15 days (*n* = 44) were predominantly slow responders who required extended stimulation beyond 15 days to reach the 18–20 mm trigger threshold. Due to the very small proportion and highly variable outcome trends (Figure 2(B,C)) of these two subgroups, reliable subgroup analysis was not feasible, and they were excluded from the primary analysis to avoid unstable estimates.

Supplementary Table 1 presents the baseline characteristics stratified by each day of Gn duration. As Gn duration increased, female age exhibited a decreasing trend, while AMH levels, BMI, and both oestradiol and progesterone levels on the day of hCG trigger showed progressively increasing trends (Supplementary Figure 1). The number of oocytes retrieved and mature oocytes also increased with longer stimulation, whereas the oocyte maturation rate exhibited a nonlinear pattern, increasing initially before plateauing after approximately 8–9 days. These systematic variations confirm that Gn duration is closely interrelated with fundamental patient characteristics and treatment response parameters. Accordingly, these factors were treated as potential confounders and included in adjusted analyses to better isolate the independent relationship between Gn duration and oocyte maturation.

### Factors associated with oocyte maturation

We evaluated factors potentially associated with oocyte maturation through several multivariable regression models with varying adjustments. All models consistently demonstrated a significant positive association between Gn duration and oocyte maturation across different models (Model 1: adjusted OR 1.07, 95% CI 1.05–1.09; Model 2: adjusted OR 1.07, 95% CI 1.05–1.09; Model 3: adjusted OR 1.07, 95% CI 1.02–1.11; Model 4: adjusted OR 1.07, 95% CI 1.05–1.09; all *p* < .01; Table 1). As shown from model 1 to model 3, female age was not significantly associated with oocyte maturation after adjusting for AMH, nor was BMI after adjusting for hCG-day hormone levels, suggesting an indirect influence of these factors on oocyte maturation.

**Table 1.** Four multivariable regression models showing the factors associated with oocyte maturation.

Variables	OR	95% CI	*p* Value
Model 1			
Gn duration (per day)	1.07	1.05–1.09	<.001***
AMH (per 1 ng/mL)	0.99	0.98–1.00	.008**
BMI (per 1 kg/m^2^)	0.99	0.99–1.00	.08**ᵗ**
Model 2			
Gn duration (per day)	1.07	1.05–1.09	<.001***
AMH (per 1 ng/mL)	0.98	0.97–0.99	<.001***
BMI (per 1 kg/m^2^)	1.00	0.99–1.01	.36
E2-HCG (per 200 pg/mL)	1.005	1.002–1.008	<.001***
P4-hCG (per 0.1 ng/mL)	0.48	0.28–0.83	.009**
Model 3			
Gn duration (per day)	1.07	1.02–1.11	.004**
Female age	1.00	0.99–1.01	.94
AMH (per 1 ng/mL)	0.98	0.96–0.99	.02*
BMI (per 1 kg/m^2^)	1.00	0.99–1.02	.80
E2-HCG (per 200 pg/mL)	1.01	1.00–1.01	.03*
P4-hCG (per 0.1 ng/mL)	0.35	0.12–1.03	.05**ᵗ**
FSH-hCG (per 1 mIU/mL)	1.00	0.98–1.02	.94
LH-hCG (per 1 mIU/mL)	1.00	0.98–1.02	.79
Model 4			
Gn duration (per day)	1.07	1.05–1.09	<.001***
AMH (per 1 ng/mL)	0.98	0.97–0.99	<.001***
E2-HCG (per 200 pg/mL)	1.01	1.00–1.01	<.001***
P4-hCG (per 0.1 ng/mL)	0.47	0.27–0.81	.007**

*Notes:* OR: odds ratio; CI: confidence interval; Gn: gonadotropin; AMH: anti‑Müllerian hormone; BMI: body mass index; E2‑hCG: oestradiol on hCG day; P4‑hCG: progesterone on hCG day; FSH‑hCG: follicle‑stimulating hormone on hCG day; LH‑hCG: luteinizing hormone on hCG day. ORs and 95% CIs were estimated using quasibinomial generalized linear models with a logit link, with the response specified as the number of MII oocytes and the number of non-MII oocytes (total retrieved oocytes minus MII oocytes). Model 1 adjusted for Gn duration, AMH and BMI; Model 2 additionally adjusted for E2‑hCG and P4‑hCG; Model 3 further adjusted for female age, FSH‑hCG and LH‑hCG; Model 4 (parsimonious) retained only variables with adjusted *p* < 0.1 in Models 1–3. ᵗ .05 ≤ *p* < .1; *p < .05; **p < .01; ***p < .001. Units for hormone levels: pg/mL for oestradiol, ng/mL for progesterone and AMH, mIU/mL for FSH and LH.

Therefore, the final parsimonious model 4 retained only the variables that were significantly associated with oocyte maturation (Gn duration, AMH, E2-hCG and P4-hCG), confirming the association of Gn duration with oocyte maturation. These analyses established that the relationship between Gn duration and oocyte maturation remains robust after accounting for various treatment characteristics in multiple models. Accordingly, subsequent analyses examining Gn duration specifically were adjusted for AMH, E2-hCG and P4-hCG to account for these potential confounding factors.

### Nonlinear relationship between Gn duration and oocyte maturation rate (OMR)

**Figure 3.**
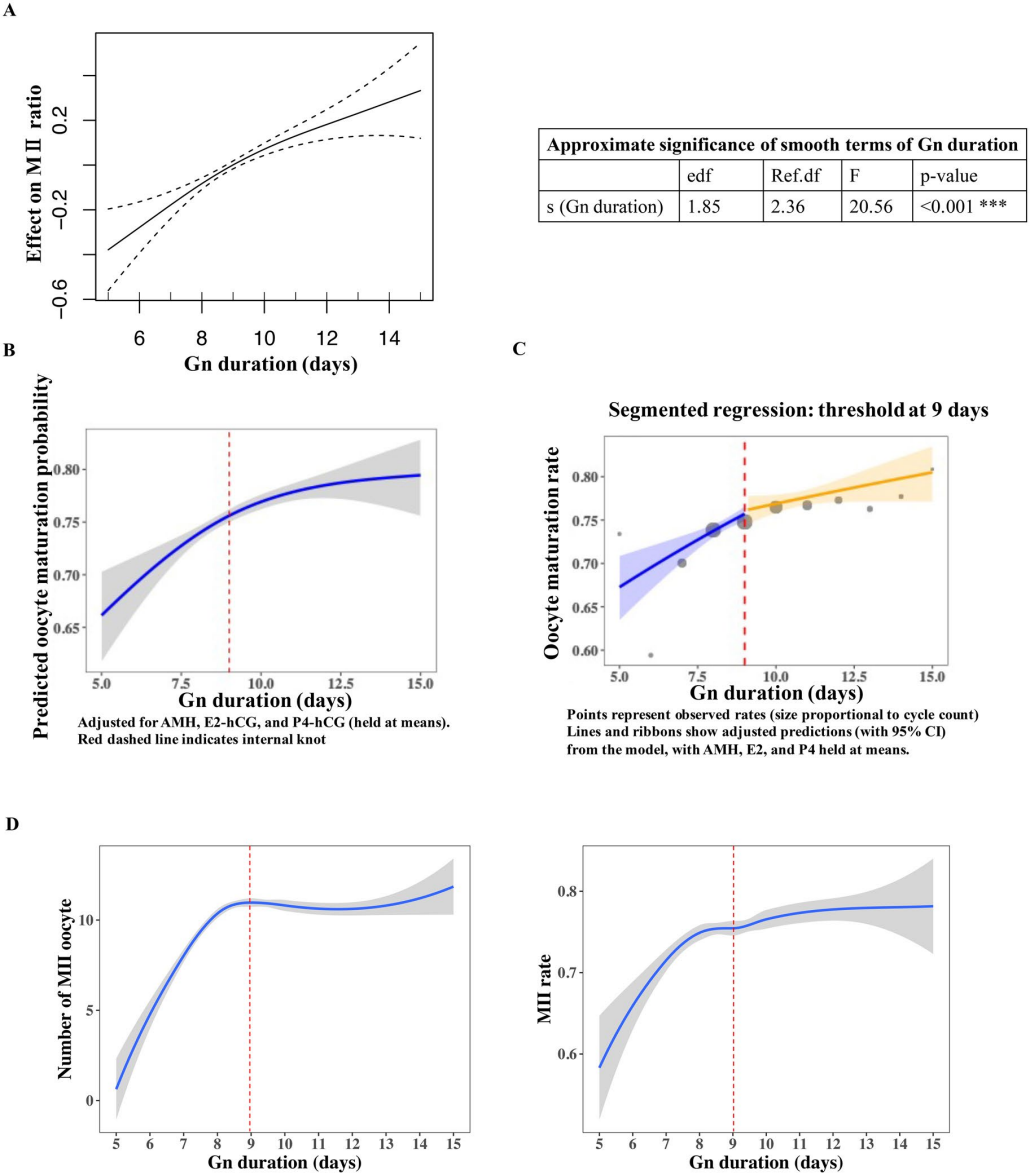
Nonlinear association between Gn stimulation duration and oocyte maturation outcomes. (A) Generalized additive model (GAM) smooth term illustrating the nonlinear relationship between Gn duration and oocyte maturation rate. (B) The natural cubic spline. (C) The segmented regression. (D) LOESS curve illustrating the relationship between Gn duration with the number of MII oocytes (left) and MII oocyte rate (right). The vertical red line indicates the threshold at 9 days, as explored using a natural cubic spline and subsequently estimated using segmented regression. The GAM, natural cubic spline and segmented regression models were adjusted for AMH, E2-hCG and P4-hCG. The LOESS curve was generated on raw data to visualize the unadjusted trends. Gn: gonadotropin; AMH: anti‑Müllerian hormone; E2: oestradiol; P4: progesterone. Units for hormone levels: pg/mL for oestradiol, ng/mL for progesterone and AMH.

We first employed a generalized additive model to assess the shape of the association between Gn duration and OMR, with adjustment for AMH, E2-hCG and P4-hCG. The smooth term for Gn duration was statistically significant (edf = 1.85, *p* < .001; Figure 3(A)), supporting a nonlinear relationship. To parametrically quantify this nonlinearity and identify a potential threshold, we fitted a natural cubic spline for Gn duration with 2 degrees of freedom within a quasibinomial logistic regression model, adjusting for the same covariables. Both spline basis terms were statistically significant (both *p* < .001), and the internal knot was located at 9 days, suggesting this as a candidate transition point (Figure 3(B)). Segmented regression analysis was then performed to estimate the association on either side of this candidate breakpoint. Below 9 days, each additional day of Gn stimulation was associated with significantly increased odds of oocyte maturation (adjusted OR 1.11, 95% CI 1.06–1.17, *p* < .001; Table 2), whereas beyond 9 days, no significant improvement was observed (adjusted OR 0.94, 95% CI 0.88–1.01, *p* = .08). Figure 3(C) provides a visual representation of this segmented relationship, displaying the observed data points and the model-fitted curves with 95% confidence intervals. The plot clearly illustrates the steep increase in maturation rate up to 9 days, followed by a plateau, supporting the threshold effect identified by the regression analysis. Finally, LOESS smoothing curves were applied to the raw data to visualize the unadjusted trends, illustrating a rapid initial increase in both the number of mature oocytes and OMR across early stimulation days, followed by a distinct plateau phase after approximately 9 days (Figure 3(D)).

**Table 2.** Segmented regression analysis of the relationship between Gn duration and oocyte maturation.

Variable	β	OR	OR 95% CI	*p* Value
Gn duration pre segment (≤9 days)	0.10	1.11	1.06–1.17	<.001***
Gn duration post segment (>9 days)	−0.06	0.94	0.88–1.01	.08**ᵗ**
Covariables				
AMH	−0.02	0.98	0.97–0.99	<.001***
E2-hCG (per 200 pg/mL)	0.01	1.005	1.002–1.008	<.001***
P4-hCG (per 0.1 ng/mL)	−0.78	0.46	0.27–0.80	.006**

*Notes:* β: regression coefficient on the logit scale; OR: odds ratio; CI: confidence interval; Gn: gonadotropin; AMH: anti-Müllerian hormone; E2-hCG: oestradiol on hCG day; P4-hCG: progesterone on hCG day. The breakpoint (9 days) was explored using a natural cubic spline and subsequently estimated using segmented regression. The model was adjusted for AMH, E2-hCG and P4-hCG. For interpretation: OR for Gn duration pre-segment represents the odds ratio per additional day before the breakpoint; OR for post‑segment represents the odds ratio per additional day after the breakpoint. ᵗ .05≤*p* < .1; **p < .01; ***p < .001. Units for hormone levels: pg/mL for oestradiol, ng/mL for progesterone and AMH.

### Subgroup analysis based on ovarian response

To evaluate potential effect modification by ovarian responsiveness, we stratified the study population into five subgroups according to the number of oocytes retrieved: 1–5, 6–10, 11–15, 16–20 and >20 oocytes. A distinct pattern was observed across response categories. In the very low responders (1–5 oocytes), no significant association was found between Gn duration and oocyte maturation (adjusted OR 1.01, 95% CI 0.94–1.08, *p* = .86; Table 3). Conversely, a consistent and statistically significant positive association was maintained across all other response subgroups (all *p* < .05). This differential pattern suggests that the relationship between Gn duration and maturation outcome is modified by the degree of ovarian response, with no observable benefit of longer stimulation durations in the very low responder population.

**Table 3.** Associations of Gn duration and oocyte maturation across ovarian response subgroups.

Subgroup	OR	95% CI	*p* Value
Number of oocytes retrieved: ≤5 group (*N* = 970)			
Gn duration (per day)	1.01	0.94–1.08	.86
AMH (per 1 ng/mL)	1.03	0.97–1.10	.37
E2-hCG (per 200 pg/mL)	1.03	0.99–1.08	.17
P4-hCG (per 1 ng/mL)	0.21	0.02–2.90	.24
Number of oocytes retrieved: 6–10 group (*N* = 2617)			
Gn duration (per day)	1.06	1.02–1.11	.004**
AMH (per 1 ng/mL)	0.95	0.93–0.98	<.001***
E2-hCG (per 200 pg/mL)	1.02	1.01–1.03	.002**
P4-hCG (per 1 ng/mL)	0.15	0.04–0.52	.003**
Number of oocytes retrieved: 11–15 group (*N* = 2490)			
Gn duration (per day)	1.07	1.03–1.12	<.001***
AMH (per 1 ng/mL)	0.98	0.96–1.008	.18
E2-hCG (per 200 pg/mL)	1.02	1.01–1.03	.003**
P4-hCG (per 1 ng/mL)	0.53	0.16–1.84	.31
Number of oocytes retrieved: 16–20 group (*N* = 1549)			
Gn duration (per day)	1.08	1.03–1.14	.002**
AMH (per 1 ng/mL)	1.00	0.97–1.02	.89
E2-hCG (per 200 pg/mL)	1.02	1.01–1.03	<.001***
P4-hCG (per 1 ng/mL)	0.65	0.18–2.46	.52
Number of oocytes retrieved: >20 group (*N* = 1667)			
Gn duration (per day)	1.08	1.03–1.13	.002**
AMH (per 1 ng/mL)	1.00	0.98–1.02	.74
E2-hCG (per 200 pg/mL)	1.01	1.00–1.01	.004**
P4-hCG (per 1 ng/mL)	0.64	0.20–2.06	.45

*Notes:* OR: odds ratio; CI: confidence interval; Gn: gonadotropin; AMH: anti-Müllerian hormone; E2-hCG: oestradiol on hCG day; P4-hCG: progesterone on hCG day. Each subgroup was analysed separately using quasibinomial generalized linear models adjusted for AMH, E2-hCG and P4-hCG (all per unit as indicated). **p < .01; ***p < .001. Units for hormone levels: pg/mL for oestradiol, ng/mL for progesterone and AMH.

### Association between Gn duration and pregnancy outcomes

This analysis was restricted to fresh embryo transfer cycles following antagonist protocols. Patients were categorized into three groups according to Gn stimulation duration: short (5–7 days), intermediate (8–10 days, defined as the threshold of 9 days ±1 day) and prolonged (≥11 days). Baseline and cycle characteristics across these groups are summarized in Table 4. Significant differences were observed in female age, BMI, AMH levels, endometrial thickness on hCG day, progesterone and oestradiol levels on hCG day, total oocytes retrieved and number of MII oocytes. However, embryo quality parameters, including the number of high-quality embryos per ET, number of embryos transferred and embryo developmental stage at transfer, showed no significant differences among groups. Biochemical pregnancy rate per ET, clinical pregnancy rate per ET and live birth rate per ET differed significantly across the duration groups (*p* < .001, *p* = .004 and *p* = .004, respectively), while miscarriage rate per CP was significantly higher in the prolonged duration group than in the intermediate duration group (16.5% vs 12.2%, *p* = .049).

**Table 4.** Baseline characteristics and pregnancy outcomes stratified by Gn duration.

Characteristics	Gn 5 –7 *N* = 383	Gn 8 –10 *N* = 3458	Gn ≥11 *N* = 646	*p*.overall	*p*.5 –7 vs 8 –10	*p*.5 –7 vs ≥11	*p*.8 –10 vs ≥11
Female age (year)	32.3(5.25)	31.8(4.82)	31.5(4.72)	.04*	.09ᵗ	.01*	.11
AMH (ng/mL)	2.76(2.22)	3.19(2.27)	3.71(3.01)	<.001***	.001**	<.001***	<.001***
BMI (kg/m^2^)	21.3(2.70)	22.4(3.00)	23.5(3.13)	<.001***	<.001***	<.001***	<.001***
Endometrial thickness on hCG day (mm)	10.3(1.88)	10.8(1.96)	11.1(2.20)	<.001***	<.001***	<.001***	.005**
E2 on hCG day (pg/mL)	1816(981)	2246(1002)	2177(1019)	<.001***	<.001***	<.001***	.13
P4 on hCG day (ng/mL)	0.52(0.25)	0.63(0.26)	0.60(0.27)	<.001***	<.001***	<.001***	.009**
No. of ET	1.70 (0.46)	1.72 (0.45)	1.71 (0.45)	.46	.31	.75	.41
No. of good embryo (per ET)	1.18 (0.91)	1.24 (0.91)	1.20 (0.93)	.27	.21	.80	.24
Embryo phase:				.45	.26	.42	.92
Cleavage	370(96.6%)	3291(95.2%)	616(95.4%)				
Blastocyst	13(3.39%)	167(4.83%)	30(4.64%)				
No. of oocyte	8.67(4.74)	11.1(4.60)	10.7(4.84)	<.001***	<.001***	<.001***	.03*
No. of MII oocytes	6.30(4.12)	8.51(4.42)	8.40(4.62)	<.001***	<.001***	<.001***	.57
Biochemical pregnancy rate (per ET)	193 (50.4%)	2128 (61.5%)	385 (59.6%)	<.001***	<.001***	.005**	.38
Clinical pregnancy rate (per ET)	166(43.3%)	1808(52.3%)	327(50.6%)	.004**	.001**	.03*	.46
Miscarriage rate (per CP)	23 (13.9%)	221(12.2%)	54 (16.5%)	.12	.57	.43	.049*
Live birth rate (per ET)	135(35.2%)	1496(43.3%)	256(39.6%)	.004**	.003**	.18	.095ᵗ

*Notes:* ET: embryo transfer; BMI: body mass index; AMH: anti‑Müllerian hormone; Gn: gonadotropin; E2‑hCG: oestradiol on hCG day; P4‑hCG: progesterone on hCG day; CP: clinical pregnancy. Continuous variables are presented as mean (*SD)* if normally distributed. Group comparisons: for continuous variables, ANOVA or Kruskal–Wallis test as appropriate; for categorical variables, χ² test or Fisher’s exact test when expected cell count <5. Post‑hoc pairwise comparisons used Tukey HSD (continuous) or χ² (categorical). ᵗ .05 ≤ *p* < .1; *p < .05; **p < .01; ***p < .001. Units for hormone levels: pg/mL for oestradiol, ng/mL for progesterone and AMH.

To examine whether the association between Gn duration and pregnancy outcomes varied by embryo developmental stage, we performed hierarchical analyses stratified by cleavage-stage (Day 3) and blastocyst-stage (Day 5/6) transfers. The results are presented in Supplementary Table 2. Among the 4277 cleavage-stage transfer cycles, the pattern was consistent with the overall findings: the intermediate Gn duration (8–10 days) was associated with the highest rates of biochemical pregnancy, clinical pregnancy and live birth, and the differences across duration groups were statistically significant (*p* < .05). In contrast, among the 210 blastocyst-stage transfer cycles (accounting for only 4.68% of all fresh transfers), the trends were directionally similar but did not reach statistical significance for pregnancy outcome, likely due to the limited sample size. Therefore, the hierarchical results should be interpreted with caution, particularly for the blastocyst stage, where the lack of statistical power precludes firm conclusions.

Considering the differences in baseline characteristics, multivariable regression analyses adjusted for female age, AMH, BMI, endometrial thickness, P4-hCG, number of good embryos transferred and embryo stage were performed to assess the independent association between Gn duration and pregnancy outcomes. The results revealed distinct associations between Gn stimulation duration and pregnancy outcomes (Table 5). Compared to the short-duration group (5–7 days), the intermediate-duration group (8–10 days) had higher odds of biochemical pregnancy (adjusted OR 1.48, 95% CI 1.17–1.88, *p* = .001), clinical pregnancy (adjusted OR 1.30, 95% CI 1.02–1.65, *p* = .03) and live birth (adjusted OR 1.28, 95% CI 1.00–1.64, *p* = .05). While prolonged Gn duration (≥11 days) was associated with a marginal increase in the odds of biochemical pregnancy (adjusted OR 1.30, 95% CI 0.97–1.74, *p* = .08), it did not confer a significant benefit for clinical pregnancy (adjusted OR 1.17, 95% CI 0.87–1.56, *p* = .30) and live birth (adjusted OR 1.06, 95% CI 0.79–1.42, *p* = .72) relative to the short-duration group.

**Table 5.** Multivariable regression analyses of the association between Gn duration and pregnancy outcomes.

Characteristic	For biochemical pregnancy			For clinical pregnancy			For live birth		
	OR	95% CI	*p* Value	OR	95% CI	*p* Value	OR	95% CI	*p* Value
Gn day group:									
5 –7	Ref			Ref			Ref		
8 –10	1.48	1.17–1.88	.001**	1.30	1.02–1.65	.03*	1.28	1.00–1.64	.05*
≥11	1.30	0.97–1.74	.08 ᵗ	1.17	0.87–1.56	.30	1.06	0.79–1.42	.72
									
Co-variables									
Female age:									
<35	Ref			Ref			Ref		
≥35	0.62	0.53–0.72	<.001***	0.60	0.51–0.70	<.001***	0.51	0.43–0.60	<.001***
AMH (per 1 ng/mL)	1.04	1.01–1.07	.005**	1.03	1.00–1.06	.02*	1.02	0.99–1.04	.25
BMI (per 1 kg/m^2^)	1.02	1.00–1.04	.07ᵗ	1.02	1.00–1.04	.07 ᵗ	1.02	1.00–1.04	.09ᵗ
Endometrial thickness on hCG day	1.08	1.04–1.11	<.001***	1.09	1.05–1.12	<.001***	1.10	1.06–1.14	<.001***
P4-hCG (per 0.1 ng/mL)	0.12	0.01–1.52	.10	0.13	0.01–1.62	.11	0.36	0.03–4.51	.43
No. of good embryo (per ET)	1.60	1.49–1.73	<.001***	1.60	1.49–1.73	<.001***	1.49	1.38–1.61	<.001***
Embryo phase at transfer:									
Cleavage	Ref			Ref			Ref		
Blastocyst	1.70	1.21–2.39	.002**	1.69	1.20–2.36	.002**	1.54	1.08–2.17	.02*

*Notes:* OR: odds ratio; CI: confidence interval; Gn: gonadotropin; AMH: anti-Müllerian hormone; BMI: body mass index; ET: embryo transfer; P4-hCG: progesterone on hCG day. ORs were estimated from multivariable logistic regression models adjusting for all variables shown in the table (female age, AMH, BMI, endometrial thickness, P4-hCG, number of good embryos, embryo phase). ᵗ .05 ≤ *p* < .1; *p < .05; **p < .01; ***p < .001. Units for hormone levels: ng/mL for progesterone and AMH.

## Discussion

### Principal findings

Our retrospective analysis demonstrates a consistent nonlinear association between gonadotropin stimulation duration and oocyte maturation rate in GnRH antagonist cycles, characterized by a plateau effect beyond around 9 days. This pattern remained robust after comprehensive adjustment for key confounders, with stimulation duration below this threshold showing a positive association with oocyte maturation odds, while extended durations beyond 9 days demonstrated diminishing returns. The stratification by ovarian response revealed important effect modification, as this association was absent in very low responders (≤5 oocytes) but maintained across all other response categories. Consistently, the 8–10 day window was associated with the highest likelihood of pregnancy and live birth, while being associated with the lowest miscarriage rate.

### Results in the context of what is known

Previous studies have reported conflicting results regarding the impact of Gn stimulation duration on IVF outcomes. Some found no significant difference in pregnancy rates across different durations [4], while others observed declining success with prolonged stimulation [13]. Variations in study design, patient populations and protocol types (e.g. agonist vs. antagonist) may explain these discrepancies. Our findings partially align with those of Yu-Chieh Yang et al. [19], who also reported differential effects of stimulation duration based on ovarian response, supporting the notion that duration should be individualized. Similarly, studies such as those by Tonko et al. [12] and Sophie Stout et al. [20] reported comparable outcomes with shorter stimulation, though not specifically in antagonist protocols. In contrast to earlier work that combined various protocols or used broader duration categories, our antagonist-specific analysis revealed a nonlinear relationship, with a distinct threshold at 9 days. This suggests that protocol-specific and response-stratified approaches are essential for accurately interpreting the role of Gn duration.

Our findings are further corroborated by recent evidence linking ovarian stimulation duration to the patient’s natural cycle length. Previous studies have demonstrated that longer menstrual cycle length is associated with more antral follicular waves and higher ovarian responsiveness, whereas shorter cycle length is a marker of ovarian ageing and poor response. Importantly, Zhao et al. [21] found that women achieving an ovarian stimulation duration to natural follicular phase length ratio between 0.67 and 0.77 obtained the highest number of oocytes and the greatest fertilization rate. For women with a normal natural follicular phase length of 12–14 days, this ratio translates into a Gn duration of 8.0–10.8 days, which corroborates our identified optimal window of 8–10 days. Therefore, the optimal Gn duration should be interpreted as a proportional reference range relative to the patient’s own natural follicular phase length, rather than an absolute fixed value.

Recent evidence has established AMH as an important predictor of individualized ovarian response. Reis et al. [22] demonstrated a significant correlation between AMH and oocyte yield, proposing cut-off values for poor (<0.72 ng/mL) and high (>4.77 ng/mL) responders. Our findings complement this evidence by showing that Gn stimulation duration remains independently associated with pregnancy outcomes even after adjusting for baseline AMH levels, suggesting that AMH assessment and dynamic monitoring of Gn duration may represent two complementary dimensions for optimizing ART outcomes.

### Clinical implications

These findings have meaningful implications for clinical decision-making. The absence of benefit from prolonged stimulation in very low responders suggests that alternative approaches, such as pretreatment optimization or initial dose modification, may represent more productive strategies than simply extending stimulation in this challenging population. In contrast, the consistent association observed in patients with ≥6 oocytes supports the value of individualized duration adjustment in these populations.

While our data suggest a plateau in maturation rate beyond 9 days, the absolute number of mature oocytes may still increase marginally with extended stimulation, albeit with diminishing returns. In such cases, a cautious extension must be carefully weighed against the potential negative impact on oocyte quality and endometrial receptivity, especially in a fresh transfer cycle. For freeze-all cycles destined for PGT, this trade-off might be different, where cautious extension of stimulation may be warranted to obtain additional mature oocytes. For cases with OHSS risk, earlier triggering may be preferable. Taken together, clinical judgement should further consider specific scenarios such as PGT cycles or cycles at higher risk of OHSS and integrate multiple dynamic indicators, including follicular growth, as well as E2 and P4 levels, to determine the appropriate timing for hCG triggering.

It is important to clarify that our findings on oocyte maturation were derived from all cycles, including those planned for PGT or freeze-all. Thus, the identified non-linear relationship and the 9-day plateau for oocyte maturation are also observed in cycles planned for PGT or freeze-all. However, the optimal 8–10 day window for pregnancy outcomes was specifically observed in fresh embryo transfer cycles, where Gn duration affects not only oocyte maturation but also endometrial receptivity through hormonal changes (E2, P4). In freeze-all cycles (including those for PGT), embryos are transferred in a subsequent unstimulated cycle, eliminating the confounding effects of ovarian stimulation on the endometrium. Consequently, clinicians may place greater emphasis on oocyte yield than on strictly targeting the 8–10-day window. However, whether prolonged stimulation affects embryo quality or ploidy remains uncertain. For PGT cycles specifically, obtaining sufficient blastocysts for testing might favour longer stimulation, but whether prolonged Gn duration affects embryo ploidy remains unknown. Future studies should evaluate whether the optimal Gn duration window for oocyte maturation translates into improved blastocyst formation or euploidy rates in freeze-all and PGT cycles.

Although patient age and hormonal profiles are known to influence ovarian response and treatment success [23,24], the association between Gn duration and pregnancy outcomes persisted after adjustment for several key confounders. The 8–10 day window emerged as particularly noteworthy, showing significantly higher odds of biochemical pregnancy, clinical pregnancy, and live birth compared to shorter durations. The attenuated association beyond 9 days, with only marginal early benefits and no significant improvement in clinical pregnancy rates and live birth rates, further supports the concept of a therapeutic window rather than a simple linear relationship. Moreover, the 8–10 day duration group has the lowest miscarriage rate while the ≥11 group has the highest miscarriage rate, with a significant difference between the two groups (Table 4).

## Strengths and limitations

This study has several limitations inherent to its retrospective design. First, selection bias may have been introduced by excluding cycles with Gn duration <5 days or >15 days and cancelled cycles. Second, information bias is possible due to reliance on medical records, although data extraction was performed with rigorous double-checking. Third, confounding bias, particularly reverse causation, cannot be excluded. While we adjusted for numerous known confounders, unmeasured factors and residual confounding remain possibilities. Specifically, we cannot exclude residual confounding from variables not captured in this retrospective analysis, including day-to-day Gn dose adjustments, use of adjunct medications (e.g. growth hormone, metformin), detailed embryo morphological parameters beyond the binary classification of good-quality versus non-good-quality (e.g. fragmentation score, blastomere symmetry, inner cell mass/trophectoderm grade for blastocysts), laboratory batch effects and clinician experience or preference. The observational design inherently limits causal inference. Future prospective studies with standardized protocols and more comprehensive covariate collection are needed to confirm the independent association between Gn duration and pregnancy outcomes.

As a single-centre study, the generalizability of our findings may be influenced by variations in patient demographics, stimulation protocols, laboratory practices and clinician preferences across different centres. For example, centres with different trigger timing strategies or embryo culture systems might observe different optimal windows. We therefore recommend that future multi-centre prospective studies be conducted to validate the 8–10 day window across diverse populations and clinical settings. Clinicians should consider their own patient characteristics and centre-specific practices when applying our findings.

Nevertheless, the large sample size, consistent findings across multiple analytical methods and biological coherence between the maturation (9-day threshold) and pregnancy (8–10 day window) observations strengthen the validity of our findings.

## Research implications

A fundamental consideration in interpreting these results is that Gn duration itself represents a treatment response parameter rather than a randomly assigned intervention. The superior outcomes observed in the 8–10 day window likely reflect, at least in part, more favourable underlying ovarian response profiles. In clinical practice, trigger timing is frequently fine-tuned according to follicular development. This 8–10 day window may represent an optimal balance between achieving adequate oocyte maturation and maintaining endometrial receptivity. Therefore, optimizing stimulation protocols to help patients achieve trigger criteria within this window (8–10 days) may help to improve reproductive outcomes. Our findings highlight the importance of tailoring the COS process to approach an ideal Gn duration window rather than rigidly targeting a fixed duration. Several strategies may help achieve this goal: (1) Individualized Gn starting regimen: Precise adjustment of the Gn start timing and Gn start dose based on comprehensive baseline assessment, including age, AMH, AFC, BMI and follicular diameter. (2) Enhanced early monitoring: Implementing earlier and more frequent ultrasound monitoring following the first Gn treatment to promptly identify aberrant follicular growth patterns, allowing for timely dose adjustments (increases for slow growth, decreases for accelerated response). (3) Terminal phase modulation: Strategic dose reduction during the final stimulation phase when multiple follicles approach maturity, potentially helping to control E2 and P4 levels and optimize endometrial environment. (4) Pretreatment optimization: Particularly for predicted poor responders, implementing appropriate pretreatment strategies to improve ovarian response before cycle initiation. This comprehensive approach to cycle management emphasizes dynamic, response-adapted stimulation rather than protocol rigidity, potentially leading to more consistent achievement of the therapeutic window associated with optimal outcomes. Based on our findings that an 8–10 day window is associated with optimal outcomes, we hypothesize that the following strategies could help achieve this window. However, these strategies require validation in future prospective studies. Building on this hypothesis, we outline several priorities for future research. First, prospective randomized controlled trials are needed to establish causality between Gn duration and reproductive outcomes. Response‑guided protocols that aim to achieve trigger criteria within the 8–10 day window, with real‑time monitoring and individualized dose adjustments, should be tested. Additionally, dedicated studies are required to evaluate whether the optimal window applies to PGT cycles and specific patient subgroups, such as women with polycystic ovary syndrome (PCOS) or diminished ovarian reserve (DOR).

## Conclusions

In conclusion, in GnRH antagonist cycles, Gn duration is associated with oocyte maturation and pregnancy outcomes following fresh embryo transfer, even after adjusting for key confounders. The 8–10 day window represents a range associated with the highest likelihood of clinical pregnancy and live birth. These findings support prioritizing dynamic, response-adapted stimulation protocols aimed at achieving trigger criteria within this window, rather than adhering to fixed duration targets.

## Supplementary Material

Supplementary material.docx

## Data Availability

The data underlying this article are available in the article. The other data are available from the corresponding author (minizenghong@126.com) on reasonable request.
