# Supplementary material for: Oocyte maturation and pregnancy outcomes in relation to gonadotropin duration in antagonist cycles
Source: Ann Med. 2026 Jul 25;58(1):2705793. doi: 10.1080/07853890.2026.2705793 (PMC13403464; doi:10.1080/07853890.2026.2705793)
Supplement: Supplementary material.docx [file IANN_A_2705793_SM1432.docx]

**Supplementary Table 1 Cycle characteristics stratified by gonadotropin stimulation duration.**

| **Gn duration (days)** | **Sample size** | **Average number of oocyte** | **Average number of M2 oocyte** | **Average rate of M2 oocyte** | **Average female age** | **Average AMH** | **Average BMI** | **Average E2 on hCG day** | **Average P4 on hCG day** |
| --- | --- | --- | --- | --- | --- | --- | --- | --- | --- |
| 1 | 3 | 1.33 | 0.67 | 0.67 | 40.67 | 0.17 | 21.95 | 224.20 | 0.18 |
| 2 | 9 | 3.22 | 2.22 | 0.85 | 36.33 | 1.63 | 24.74 | 1187.69 | 0.29 |
| 3 | 7 | 2.00 | 1.43 | 0.71 | 35.14 | 0.46 | 21.56 | 587.30 | 0.67 |
| 4 | 16 | 3.69 | 2.94 | 0.74 | 39.69 | 1.46 | 22.52 | 612.52 | 0.34 |
| 5 | 35 | 3.11 | 2.29 | 0.61 | 35.60 | 0.86 | 21.66 | 936.59 | 0.51 |
| 6 | 63 | 5.98 | 3.56 | 0.62 | 34.22 | 2.32 | 21.26 | 1468.87 | 0.49 |
| 7 | 587 | 11.19 | 7.83 | 0.72 | 32.36 | 3.35 | 21.68 | 2230.45 | 0.64 |
| 8 | 2622 | 14.03 | 10.36 | 0.75 | 31.55 | 3.81 | 22.03 | 2787.36 | 0.75 |
| 9 | 2861 | 14.67 | 10.97 | 0.75 | 31.88 | 3.81 | 22.41 | 3008.39 | 0.83 |
| 10 | 1739 | 14.13 | 10.81 | 0.77 | 31.91 | 3.86 | 22.75 | 2890.56 | 0.86 |
| 11 | 795 | 13.64 | 10.46 | 0.77 | 31.78 | 3.83 | 23.28 | 2822.72 | 0.88 |
| 12 | 339 | 13.82 | 10.68 | 0.77 | 31.16 | 4.17 | 23.53 | 2658.49 | 0.87 |
| 13 | 153 | 12.95 | 9.88 | 0.77 | 31.99 | 4.41 | 24.09 | 2525.76 | 0.87 |
| 14 | 70.00 | 16.03 | 12.46 | 0.79 | 30.14 | 5.72 | 24.02 | 3334.92 | 0.83 |
| 15 | 29 | 13.86 | 11.21 | 0.78 | 30.76 | 6.84 | 23.75 | 3492.12 | 1.39 |
| 16 | 19 | 14.63 | 11.42 | 0.75 | 30.32 | 6.23 | 23.52 | 3386.35 | 1.28 |
| 17 | 11 | 11.09 | 7.55 | 0.69 | 29.36 | 8.14 | 24.43 | 2625.45 | 0.50 |
| 18 | 9 | 12.89 | 9.33 | 0.79 | 29.44 | 5.68 | 23.14 | 3021.67 | 0.88 |
| 19 | 3 | 20.33 | 18.67 | 0.91 | 31.00 | 6.62 | 23.42 | 5424.33 | 0.70 |
| 20 | 1 | 36.00 | 30.00 | 0.83 | 29.00 | 23.00 | 23.63 |  |  |
| 26 | 1 | 4.00 | 3.00 | 0.75 | 28.00 | 2.12 | 23.63 | 1018.00 | 0.50 |

**Supplementary Figure 1 Associations between Gn duration and patients characteristics or hormonal levels: female age, AMH level, BMI, E2-hCG, and P4-hCG.**


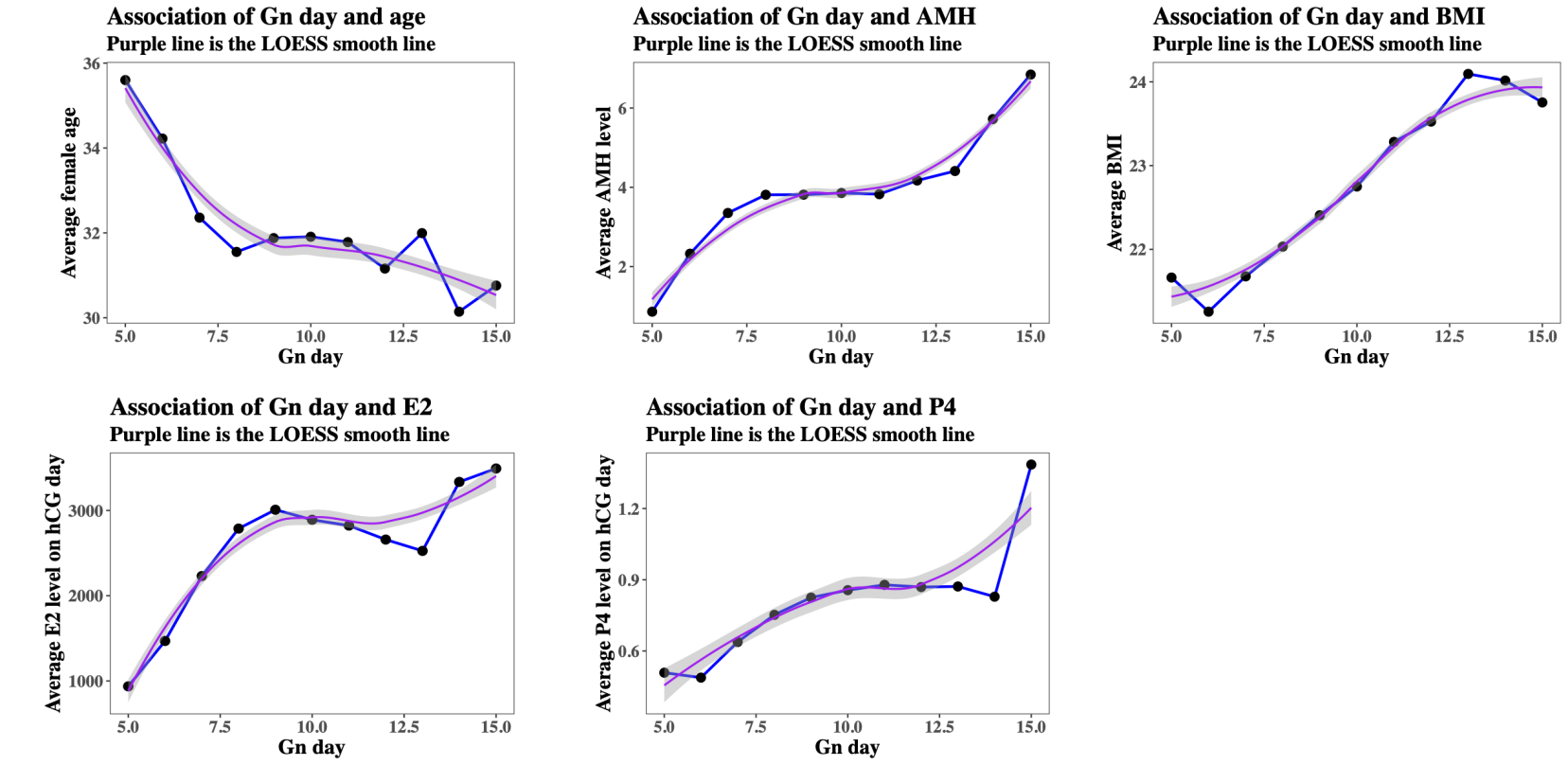
BMI, body mass index; AMH, anti‑Müllerian hormone; Gn, gonadotropin; E2‑hCG, estradiol on hCG day; P4‑hCG, progesterone on hCG day. Units for hormone levels: pg/mL for estradiol, ng/mL for progesterone and AMH.

**Supplementary Table 2 Pregnancy outcomes stratified by Gn duration and embryo developmental stage (cleavage-stage vs. blastocyst-stage transfers).**

**Panel A Cleavage-stage embryo transfer**

|  | **Gn 5~7**  **N=370** | **Gn 8~10**  **N=3291** | **Gn >= 11**  **N=616** | **p.overall** | **p.5~7 vs 8~10** | **p.5~7 vs >=11** | **p.8~10 vs >=11** |
| --- | --- | --- | --- | --- | --- | --- | --- |
| Female age (year) | 32.3(5.27) | 31.8(4.84) | 31.4(4.68) | 0.03 * | 0.19 | 0.03 * | 0.17 |
| AMH (ng/ml) | 2.72(2.19) | 3.18(2.26) | 3.66(3.00) | <0.001 *** | 0.001 *** | <0.001 *** | 0.001 ** |
| BMI (kg/m^2^) | 21.3(2.70) | 22.4(2.99) | 23.5(3.16) | <0.001 *** | <0.001 *** | <0.001 *** | <0.001 *** |
| Endometrial thickness on hCG day (mm) | 10.4(1.88) | 10.8(1.95) | 11.2(2.20) | <0.001 *** | <0.001 *** | <0.001 *** | 0.006 ** |
| E2 on hCG day (pg/ml) | 1800(969) | 2237(998) | 2166(1016) | <0.001 *** | 0.000 *** | <0.001 *** | 0.28 |
| P4 on hCG day (ng/ml) | 0.51(0.25) | 0.63(0.26) | 0.60(0.27) | <0.001 *** | 0.000 *** | <0.001 *** | 0.03 * |
| No of ET | 1.72(0.45) | 1.76(0.43) | 1.74(0.44) | 0.21 | 0.25 | 0.74 | 0.64 |
| No of good embryo (per ET) | 1.22(0.90) | 1.30(0.89) | 1.25(0.91) | 0.13 | 0.20 | 0.83 | 0.41 |
| No of oocyte | 8.44(4.53) | 11.0(4.48) | 10.5(4.67) | <0.001 *** | 0.000 *** | 0.000 *** | 0.05 * |
| No of MII oocytes | 6.12(3.94) | 8.37(4.34) | 8.23(4.52) | <0.001 *** | <0.001 | <0.001 *** | 0.76 |
| Biochemical pregnancy rate (per ET) | 189(51.1%) | 2026(61.6%) | 368(59.7%) | <0.001 *** | <0.001 *** | 0.01 * | 0.42 |
| Clinical pregnancy rate (per ET) | 163(44.1%) | 1720(52.3%) | 315(51.1%) | 0.01 * | 0.01 * | 0.05 * | 0.64 |
| Miscarriage rate (per CP) | 22(13.50%) | 213(12.38%) | 51(16.19%) | 0.18 | 0.68 | 0.44 | 0.07 ᵗ |
| Live birth rate (per ET) | 133(35.9%) | 1421(43.2%) | 247(40.1%) | 0.02 * | 0.03 * | 0.23 | 0.22 |

**Panel B Blastocyst-stage embryo transfer**

|  | **Gn 5~7**  **N=13** | **Gn 8~10**  **N=167** | **Gn >= 11**  **N=30** | **p.overall** | **p.5~7 vs 8~10** | **p.5~7 vs >=11** | **p.8~10 vs >=11** |
| --- | --- | --- | --- | --- | --- | --- | --- |
| Female age (year) | 32.4(4.93) | 32.2(4.29) | 32.8(5.48) | 0.87 | 0.99 | 0.97 | 0.86 |
| AMH (ng/ml) | 3.80(2.91) | 3.40(2.30) | 4.71(3.14) | 0.11 | 0.88 | 0.63 | 0.09 ᵗ |
| BMI (kg/m^2^) | 21.1(2.82) | 22.4(3.17) | 23.9(2.60) | 0.007 ** | 0.32 | 0.02 * | 0.02 * |
| Endometrial thickness on hCG day (mm) | 10.1(2.17) | 10.6(2.07) | 10.1(1.90) | 0.39 | 0.71 | 1.00 | 0.42 |
| E2 on hCG day (pg/ml) | 2382(1247) | 2471(1078) | 2439(1065) | 0.97 | 0.97 | 0.99 | 0.99 |
| P4 on hCG day (ng/ml) | 0.66(0.24) | 0.65(0.25) | 0.61(0.24) | 0.76 | 0.99 | 0.85 | 0.75 |
| No of ET | 1.00 [1.00;1.00] | 1.00 [1.00;1.00] | 1.00 [1.00;1.00] | 0.23 | 0.25 | 0.25 | 0.46 |
| No of good embryo (per ET) | 0.00 [0.00;0.00] | 0.00 [0.00;0.00] | 0.00 [0.00;0.00] | 0.13 | 0.19 | 0.19 | 0.55 |
| No of oocyte | 15.1(6.13) | 14.7(5.56) | 15.0(6.11) | 0.95 | 0.98 | 1.00 | 0.97 |
| No of MII oocytes | 11.3(5.79) | 11.2(5.16) | 11.7(5.41) | 0.88 | 1.00 | 0.97 | 0.87 |
| Biochemical pregnancy rate (per ET) | 4(30.8%) | 102(61.1%) | 17(56.7%) | 0.10 ᵗ | 0.19 | 0.33 | 0.80 |
| Clinical pregnancy rate (per ET) | 3(23.1%) | 88(52.7%) | 12(40.0%) | 0.07 ᵗ | 0.23 | 0.49 | 0.42 |
| Miscarriage rate (per CP) | 1(33.3%) | 8(9.09%) | 3(25.0%) | 0.15 | 0.25 | 0.48 | 0.13 |
| Live birth rate (per ET) | 2(15.4%) | 75(44.9%) | 9(30.0%) | 0.05 * | 0.22 | 0.46 | 0.28 |

Note: ET, embryo transfer; BMI, body mass index; AMH, anti‑Müllerian hormone; Gn, gonadotropin; E2‑hCG, estradiol on hCG day; P4‑hCG, progesterone on hCG day; CP, clinical pregnancy. Continuous variables are presented as mean ± SD if normally distributed; † variables with non‑normal distribution are presented as median (IQR). Group comparisons: for continuous variables, ANOVA or Kruskal‑Wallis test as appropriate; for categorical variables, χ² test or Fisher’s exact test when expected cell count <5. Post‑hoc pairwise comparisons used Tukey HSD (continuous) or χ² (categorical). ᵗ 0.05≤p<0.1; * p<0.05; ** p<0.01; *** p<0.001. Units for hormone levels: pg/mL for estradiol, ng/mL for progesterone and AMH.
